# New Active Ingredients for Sustainable Modern Chemical Crop Protection in Agriculture

**DOI:** 10.1002/cssc.202401042

**Published:** 2024-10-07

**Authors:** Peter Jeschke

**Affiliations:** ^1^ Institute of Organic Chemistry and Macromolecular Chemistry Heinrich Heine University Düsseldorf University Street 1 D-40225 Duesseldorf Germany

**Keywords:** Active ingredient, Crop protection, Agrochemical, Chiral, Halogen, Sustainability

## Abstract

Today, the agrochemical industry faces enormous challenges to ensure the sustainable supply of high‐quality food, efficient water use, low environmental impact, and the growing world population. The shortage of agrochemicals due to consumer perception, changing needs of farmers and ever‐changing regulatory requirements is higher than the number of active ingredients that are placed on the market. The introduction of halogen atoms into an active ingredient molecule offers the opportunity to optimize its physico‐chemical properties such as molecular lipophilicity. As early as 2010, around four‐fifths of modern agrochemicals on the market contained halogen atoms. In addition, it becomes clear that modern agrochemicals have increasingly complex molecular structures with one or more stereogenic centers in the molecule. Today, almost half of modern agrochemicals are chiral molecules (herbicides, insecticides/acaricides/nematicides ≪ fungicides) and most of them consist of mixtures such as racemic mixtures of enantiomers, followed by mixtures of diastereomers and mixtures of pure enantiomers. Therefore, it is important that halogen‐containing substituents or stereogenic centers are considered in the structural optimization of the active ingredients to ultimately develop sustainable agrochemicals in terms of efficacy, ecotoxicology, ease of use and cost‐effectiveness.

## Graphical abstract

To ensure sustainability in crop protection, development of modern agrochemicals with improved selectivity, efficacy and environmental profile is essential. This ambitious goal can be achieved, for example, by considering halogen‐containing motifs or sterogenic centers in an active ingredient, having a significant influence on its physico‐chemical properties. For this reason, a perspective is given on sustainability in crop protection, based on a recently published chapter on the topic crop protection in the Ullmann′Encyclopedia of Industrial Chemistry.

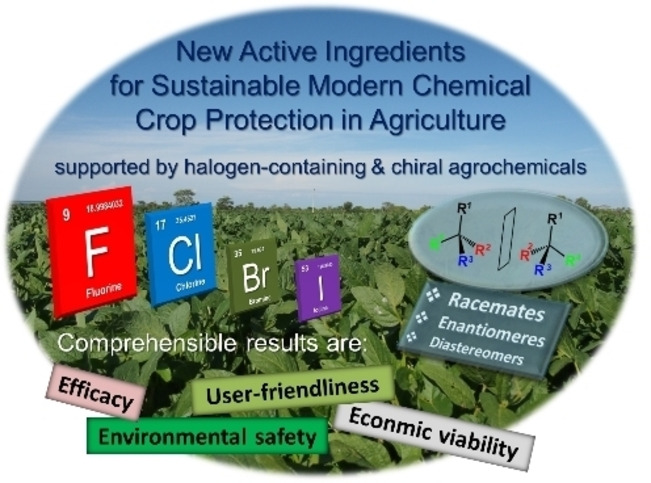

## 1 Introduction

To ensure sustainability in crop protection in the long term, the modern agrochemical industry faces enormous challenges. These result from the provision of high‐quality food, increasing water consumption, environmental pollution and even a growing world population. The agricultural methods range from soil preparation to the protection of the harvest itself and extend to its transport and storage and all of these must meet the criterion of sustainability and cost efficiency. Today, sustainability in crop protection also refers to tailor‐made approaches that increase farmers’ productivity and efficiency while minimizing the environmental impact of agriculture. Modern technologies such as seed treatment, formulation types, adjuvants and drip irrigation support the technical application of active ingredients (a.i.s).^[1]^

Chemical crop protection is one of the most cost‐effective methods of crop protection, as it supports high yields and ensures stable and sustainable growth at any time of the year. In addition, resistance management is essential for sustainable and worldwide control of weeds, plant diseases and insect pests.

However, the increasing loss of established a.i.s due to consumer perception, changing grower needs and everchanging regulatory requirements is higher than the number of agrochemicals being introduced into the crop protection market annually. To ensure sustainability in modern crop protection, the development of novel, innovative agrochemicals is mandatory for the continuous improvement of the selectivity, efficacy, and favorable environmental profile of a.i.s.

In this context, for example, the introduction of halogen atoms and halogen‐containing motifs into a molecule is an important tool for influencing its physico‐chemical properties.^[2]^ At the same time, the strategy to achieve the ambitious goals is also to take advantage of the unique properties of molecules containing stereogenic centers. In the past, numerous natural products and their congeners have been an important source of inspiration for the design of new a.i.s. However, the molecular structures of the resulting compounds have become increasingly complex and require the use of modern synthesis methods.[^3^, ^4^]

## 2 Challenges for Modern Agrochemicals

As an integral part of complex ecological and economic systems, the aim of crop protection from a business and ecological point of view is to avoid pre‐harvest losses, i. e. ensure sustainable crop yields with high quality, to keep plants healthy, replace labour‐intensive methods and contributing to the development of new agricultural production despite the constraints of climate or soil.

In crop protection, suitable strategies and modern agrochemicals are used that provide long‐term protection against various weeds, plant diseases and insect pests. In integrated pest management on a global level, for example, the efficient control of residual pests with minimal use of carefully selected modern agrochemicals is important. This means that farmers take a holistic approach and apply measures that limit the use of modern agrochemicals to the necessary minimum. To achieve this, current and future crop protection products must meet these high requirements, which are essential for their worldwide application.

In the search for new a.i.s, halogen atoms or halogenated substituents are still very important for their structural optimization to develop optimal agrochemical products in terms of efficacy, exotoxicology, user‐friendliness and economic viability. In addition, modern agrochemicals have been shown to possess more sophisticated molecular structures with one or more stereogenic centers in the molecule. Therefore, natural products and their congeneric lead structures are also a sustainable source of inspiration for the development of chiral and enantiomerically pure a.i.s. It is well known that the metabolism of an a.i. is characterized by the substitution pattern or respective stereoisomers, soil stability and water solubility. In particular, the influence of halogen substituents on physico‐chemical properties such as molecular lipophilicity (e. g. key parameters for absorption, distribution, metabolism, and excretion; ADME)^[5]^ can be emphasized.

Therefore, both optimization techniques are important for the discovery and development of a modern agrochemical. The following two sections illustrate this promising, unified approach.

### 2.1 Active Ingredients with Halogen‐Containing Motifs

Since 2016, around 77 % of the marketed modern agrochemicals (the total number was 39 commercial products) are halogen‐substituted (nematicides, herbicides ≪ insecticides/acaricides, fungicides) and a significant rise of fluorine‐containing products has been observed. For example, the number of agrochemicals containing fluorine atoms has increased significantly (~64 %).^[6]^

Outstanding progress has been made in industrial‐scale technical manufacturing for large quantities of important fluorine‐substituted building blocks, such as the 3‐(difluoromethyl)‐1‐methyl‐1*H*‐pyrazole‐4‐carboxylate (DFMMP), which is used to produce fungicidal succinate dehydrogenase inhibitors (SDHIs).[^7^, ^8^]

**Figure 1:**
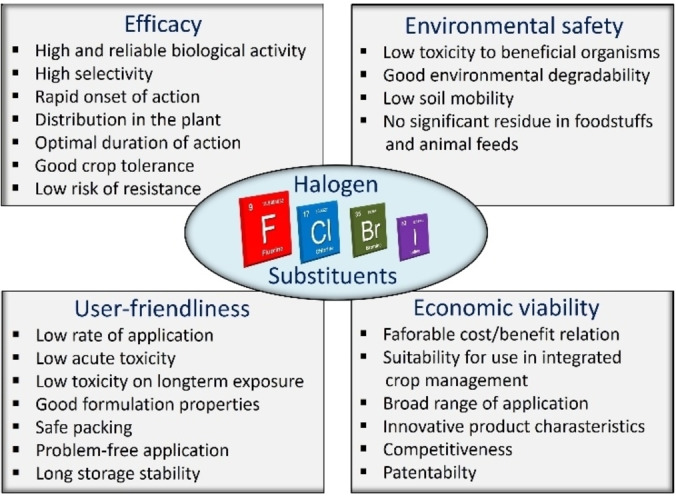
Search for the optimal agrochemical product in modern crop protection using halogen substituents; see Ref. [9].

Therefore, halogenated a.i.s have been able to play a significant role in the development of modern and innovative agrochemicals over the past 40 years (Figure 1).[^9^, ^10^, ^11^]

The importance of halogen atoms and/or halogenated substituents can be attributed to the well‐known steric effects (e. g., carbon‐halogen bond lengths, van der Waals radii),^[12]^ electronic effects (e. g., electronegativities of halogens and halogenated groups on the Pauling scale)[^13^, ^14^] the polarity of the carbon‐halogen bond (e. g., dipole moments, drug‐receptor interaction)^[15]^ and pK_a_ value effects (e. g., H‐bond, interaction with the target). In addition, metabolic, oxidative, and thermal stability (carbon‐halogen binding energy, metabolic stabilization by electron withdrawal, halogenated groups)[^16^, ^17^] can be improved.

The influence of halogen atoms is illustrated by biological activity.^[1]^ In this context, the impact of halogen atoms or halogenated substituents in a.i.s can lead to an improvement or to a decline in biological activity. Depending on the appropriate target, the ligand‐target binding interactions, or mode of action (MoA), can be influenced by the physico‐chemical property profile of the molecules being tested. About 52 % of current fluorinated agrochemicals contain a trifluoromethyl group (F_3_C), about 20 % contain a difluoromethyl group (F_2_HC), and about 4 % contain a trifluoromethoxy group (F_2_HC−O) as a substituent on the phenyl or heterocyclic moieties. This significant increase in halogen‐substituted agrochemicals [Br/Cl (3 %), Br/F (3 %)<Cl (13 %)<F/Cl (40 %)=F (40 %)] illustrates the continued importance of halogen atoms or halogenated substituents in modern crop protection products.^[6]^ The fact that there are currently only a few iodine‐containing pesticides is due to the very expensive production of iodine‐substituted intermediates.

Preferred heterocyclic agrochemicals containing halogen atoms or halogenated motifs are currently pyrazoles, followed by pyridines, pyrimidines or 3,6‐dihydro‐2,6‐dioxo‐1(2*H*)‐pyrimidines and the bicyclic part such as quinoline.^[2]^

With the latest generation of halogenated agrochemicals, two new and two previously unknown MoAs for fungicides, three new MoAs for insecticides/acaricides and four new classes (new subgroups of the Insecticide Resistance Action Committee (IRAC; an Expert Committee of Crop Life)) for known MoAs have been identified. Finally, three new MoAs were established for nematode control.^[2]^

However, it has been shown that the success of halogenated agrochemicals is not unlimited. Therefore, over the past 10 years, the agrochemical industry has been searched for halogen‐free a.i.s for various crop protection applications that meet regulatory requirements. But in general, there is no doubt that halogen atoms and halogen‐containing substituents will remain important in the future to efficiently modulate the properties of a.i.s.

### 2.2 Active Ingredients Containing Stereogenic Centers

**Figure 2:**
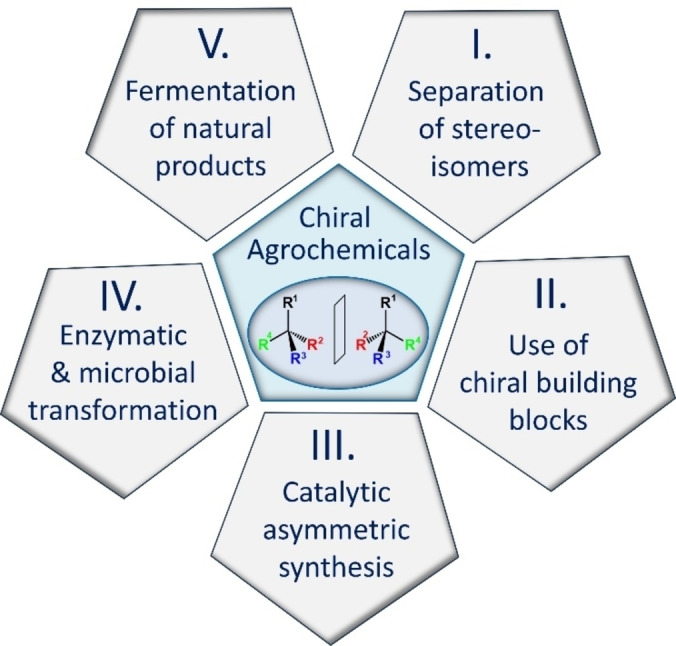
Various technical manufacturing methods for the preparation of chiral modern agrochemicals; see Ref. [3].

Of the agrochemicals introduced to the market in the period 2010–2020, around 48 % of the products are chiral molecules (herbicides, insecticides/acaricides/nematicides ≪ fungicides) and most of them are mixtures such as racemic mixtures of enantiomers (56 %), mixtures of diastereomers (22 %) and mixtures of pure enantiomers (22 %). To enable the synthesis of modern chiral agrochemicals with the required efficiency, the technical manufacturing methods I to V (Figure 2) are used, which allow synthesis to be carried out on an industrial scale:

- Separation of stereoisomers: Chiral resolution by crystallization in the presence of a chiral resolving reagent (chiral auxiliary), is enormous important in the manufacturing process of intermediates for agrochemicals. In this case, the chiral reagent must not be too expensive, must be available in its both enantiomeric forms and should contribute to a high enantiomer excess. A limitation in practice is the possible increase in impurities and tolerance to them in the large‐scale crystallization process. In recent decades, countercurrent chromatography (CCC) has been successfully used in the field of chiral separations due to its low solvent consumption and easy scale‐up.
- Use of chiral building blocks: The chiral pool contains inexpensive, commercially available chiral natural products (carbohydrates, terpenes, alkaloids, hydroxyl‐ and amino acids), that are already present with high enantiomeric excess (*ee*‐value) and can therefore be used as intermediates (Table 1
  **Table 1:** Selected agrochemicals manufactured from chiral pool intermediates.

Common name	Chiral pool	Launch year	Use	Mode of action/target^[b]^
Deltamethrin	δ‐3‐Carene	1977	Insecticide	Sodium channel
Tau‐fluvalinate	(*R*)‐Valine	1984	Insecticide^[a]^	ACCase
Haloxyfop‐P‐methyl	(*S*)‐Lactic acid	1994	Herbicide	ACCase
Iprovalicarb	(*S*)‐Valine	1998	Fungicide	Cellulose synthase

[a] Incl. acaricide. [b] ACCase: Acetyl‐CoA carboxylase; see Ref. [3]
).
- Catalytic asymmetric synthesis: The use of chiral catalysts to transfer and amplify chirality in chemical reactions is a central topic in modern agrochemistry. Hereby three different kinds of chiral catalysts are of interest: (a) metal‐ligand complexes derived from chiral ligands, (b) chiral organo‐catalysts, and (c) biocatalysts. Numerous homogeneous, chiral, transition‐metal catalysts, particularly organometallic catalysts, known that can be used in industrial large‐scale manufacturing processes such as asymmetric hydrogenations, Sharpless epoxidation and dihydroxylation steps, asymmetric cyclopropanation, and isomerization process‐ses. A significant example is the development of a catalytic homogenous enantioselective hydrogenation process (hydrogen, Ir‐Josiphos catalyst) for the (*S*)‐configurated arylamine precursor used in the technical preparation of the grass herbicide (*S*)‐metolachlor (around 80% ee‐value). This is the largest catalytic asymmetric transformation process on a multiple‐ton scale (> 10,000 tons/year) run today.^[18]^ Despite the advances made in catalytic‐asymmetric processes over the past decade, few agrochemicals are produced on an industrial scale in enantiomerically pure or enriched form.
- Enzymatic and microbial transformations: Large‐scale applicable biocatalytic processes have been developed to produce (*S*)‐amino acids in technical quantities. In this context enzyme‐based resolution procedures for amino acids via hydrolyses of their esters are important. These have been achieved, e.g., by using proteases (α‐chymotrypsin, subtilisin, and other microbial proteases, and sulfhydryl proteases of plant origin) and lipases. The basic principle commercially applied methods cover resolution processes is the enzymatic resolution of racemates and asymmetric (bio)catalytic conversion starting from prochiral compounds. Examples of enzymatic resolution on an industrial scale are acylase‐, amidase‐, hydantoinase‐, and ß‐lactam hydrolase‐mediated production of (*S*)‐amino acids such as Ala, Phe, or Met,[^19^, ^20^] or the enzyme‐catalyzed synthesis of a key intermediate for the (*R*)‐enantiomer of the fungicide metalaxyl.^[20]^ The use of biocatalysts during the enzymatic synthesis of agrochemicals is also considered to be a contribution to ‘green chemistry’, as demonstrated for the grass herbicide (*S*)‐metolachlor.^[21]^ In the past decades, whole‐cell biocatalyst using engineered *Escherichia coli* seems to be the most promising method for large‐scale and low‐cost production.^[22]^
- Fermentation of natural products: Some secondary metabolites used as agrochemicals can be produced by industrial fermentation efficiently (Table 2
  **Table 2:** Selection of natural products used as agrochemicals obtained by fermentation in the presence of powerful strains.

Common name	Strain	Launch year	Use	Mode of action/ Target^[b]^
Validamycin	*Streptomyces hygroscopicus* *var. limoneus*	1972	Fungicide	Protein synthesis
Bialaphos	*Streptomyces hygroscopicus*	1984	Herbicide	GluS
Milbemectin B	*Streptomyces hygroscopicus* subsp. *aure‐olacrimosus*	1990	Insecticide^[a]^	GluCl‐channel
Spinosad	*Saccharopolyspora spinosa*	1997	Insecticide	*n*AChR

[a] Incl. acaricide and nematicide. [b] GluS: Glutamine synthase; GluCl‐channel: Glutamate‐gated chloride channel; *n*AChR: Nicotinic acetylcholine receptor; see Ref. [3]
).

For example, older aminoglycoside antibiotic fungicides are effective in controlling certain plant pathogens: (a) validamycin, suitable for the control of *Rhizoctonia solani* in rice, potatoes, vegetables, and other crops, isolated from the strain *Streptomyces hygroscopicus* var. *limoneus*. The tripeptide bialaphos (syn. bilanaphos), developed as the first herbicide produced by microorganism fermentation (*S. hygroscopicus*), is a non‐selective foliar‐applied herbicide with strong herbicidal activities against a wide range of mono‐ and dicotyledonous weeds. Furthermore, various 16‐membered macrocyclic lactone insecticides, acaricides, and nematicides such as milbemectin B can be prepared by a large‐scale fermentation of *Streptomyces hygroscopicus* subsp. *aureolacrimosus* strains, respectively. Finally, the bioinsecticides spinosyns are produced by the actinomycete *Saccharopolyspora spinosa*.

The selection of the appropriate technical manufacturing methods I to V for the preparation of the chiral agrochemicals depends on the chiral pool of starting materials or the availability of microorganisms (strains) useful for efficient fermentation of final product or its precursors in good yield, which finally influences the total production costs of the industrial large‐scale manufacturing process remarkably. In the future, the progressive expansion of biocatalytic pathways, which include both enzymatic dissolutions and asymmetric (bio)catalysis, will be the focus of the synthesis of modern agrochemicals useful for sustainable crop protection.

For a reliable risk assessment, it is very important to understand the variability of the effects of stereoisomers of chiral modern agrochemicals. Nevertheless, due to the need for cost‐efficient asymmetric technologies, most chiral modern agrochemicals will continue to be produced mainly as racemates in the coming years.

To learn more about sustainability in modern crop protection, it is recommended to read the updated chapter in Ullmann's and all references cited therein^[23]^ and for some additional background information, please refer to the editorial in this issue.^[24]^

## Conflict of Interests

The authors declare no conflict of interest.
